# In-Situ Monitoring of a Filament Wound Pressure Vessel by the MWCNT Sensor under Hydraulic Fatigue Cycling and Pressurization

**DOI:** 10.3390/s19061396

**Published:** 2019-03-21

**Authors:** Biao Xiao, Bin Yang, Fu-Zhen Xuan, Yun Wan, Chaojie Hu, Pengcheng Jin, Hongshuai Lei, Yanxun Xiang, Kang Yang

**Affiliations:** 1 School of Mechanical and Power Engineering, East China University of Science and Technology, Shanghai 200237, China; xiaobiao@ssei.cn (B.X.); y20170063@mail.ecust.edu.cn (C.H.); yxxiang@ecust.edu.cn (Y.X.); yangkangcxm@163.com (K.Y.); 2 School of Civil Engineering and Architecture, East China Jiaotong University, Nanchang 330013, China; wanyun0505@163.com; 3 Chinese Society for Composite Materials, Beijing 100191, China; jinpengcheng@csfcm.org; 4 Beijing Key Laboratory of Lightweight Multi-functional Composite Materials and Structures, Beijing Institute of Technology, Beijing 100081, China; lei123shuai@126.com

**Keywords:** pressure vessels, MWCNT sensor, fatigue cycling, bursting, structural health monitoring

## Abstract

As a result of the high specific strength/stiffness to mass ratio, filament wound composite pressure vessels are extensively used to contain gas or fluid under pressure. The ability to in-situ monitor the composite pressure vessels for possible damage is important for high-pressure medium storage industries. This paper describes an in-situ monitoring method to permanently monitor composite pressure vessels for their structural integrity. The sensor is made of a multi-walled carbon nanotube (MWCNT) that can be embedded in the composite skin of the pressure vessels. The sensing ability of the sensor is firstly evaluated in various mechanical tests, and in-situ monitoring experiments of a full-scale composite pressure vessel during hydraulic fatigue cycling and pressurization are performed. The monitoring results of the MWCNT sensor are compared with the strains measured by the strain gauges. The results show that the measured signal by the developed sensor matches the mechanical behavior of the composite laminates under various load conditions. In the hydraulic fatigue test, the relationship between the resistance and the strain is built, and could be used to quantitative monitor the filament wound pressure vessel. The bursting of the pressure vessel can be detected by the sharp increase of the MWCNT sensor resistance. Embedding the MWCNT sensor into the composite pressure vessel is successfully demonstrated as a promising method for structural health monitoring.

## 1. Introduction

Filament wound pressure vessels have been extensively used in many engineering fields such as in the nuclear energy, naval, petrochemical, and aero-space industries [1,2]. Because of the complex working environment, the safety of in-service filament wound pressure vessels has drawn increasing attention [3,4]. Variable pressurization occurs when the medium is being used and then replenished, and this is analogous to the fatigue loading of the pressure vessel. Damages in the composite skin are a major and frequently occurring failure that tremendously affect the strength, stiffness, and usable service life of a composite pressure vessel [5]. Studies have shown that composite pressure vessels eventually fail when damages in the laminate skin reach the maximum damage limit [6]. Hence, the detection of damages in the composite skin is critical for safe and reliable implementation in the structural integrity assessment of filament wound pressure vessels.

Because the detection of filament wound pressure vessels is of fundamental importance in order to avoid the occurrence of an abrupt and catastrophic event, studies related to the detection of damages in the pressure vessels are of notable interests [6,7,8,9,10,11]. To ensure the service safety of the pressure vessels, several non-destructive (NDT) methods have been used to monitor the initiation and development of damage in composite pressure vessels [12,13]. Generally, the NDT methods can be divided into two categories, namely: passive safety systems, such as the vibration-based structural damage detection technique and the acoustic emission (AE) [5,11,14,15,16], and active safety systems, such as the strain monitoring using bragg grating optical fiber sensors [9], guided waves [17,18], and nano-material-based sensors [19,20]. Vibration-based structural damage detection is based on the fact that damage occurred in a pressure vessel would result in changes in the structural dynamic characteristics, and the variation of the physical properties could be identified [21]. However, vibration-based methods often require special loading or handling conditions during inspection. Therefore, it is only suitable for periodic inspections rather than continuous monitoring. In the AE analysis of a filament wound pressure vessel, it is often difficult to distinguish the AE signals, because the damage events generally have similar acoustic properties [6]. Optical fibers can be employed as a monitoring system throughout manufacturing, transport, installation, and the service life of the pressure vessel, but the handling of fragile optical fibers is difficult [22,23,24]. In recent years, guided waves have gained prominence for damage detection because of their potential for in-service structural health monitoring (SHM) and inspection at inaccessible locations [25]. However, the post-processing of guided wave signals is a challenge. In our previous papers [19,20], we continually monitored the health state of glass fiber (GF)/epoxy composite laminates via coating multi-walled carbon nanotube (MWCNT) on fiber surfaces. The principle of this electrical resistance measurement is that the conductive networks can break up with the appearance of damage in composites. Compared with other NDT methods, nano-material-based sensors do not involve the attachment of external sensors or additional fibers input in the structures [26]. Zhang et al. [27] described the development of a hierarchically engineered micro-nano hybrid composite system. They used a solvent assisted dispersion or spread method to disperse the CNTs in composites, and the in-situ damage sensing of composite laminates upon loading was presented. Chowdhury et al. [28] addressed the piezoresistive sensing mechanism experimentally. Thostenson et al. [29] reported the pioneering work of self-sensing composites using CNTs in early 2000. They examined the research work reported in the literature on the structure and processing of cCNTs, as well as the characterization and property modeling of carbon nanotubes and their composites. Although the resistance-based nano sensors are verified to have a high sensitivity in SHM [19,30], research on monitoring the failure in the filament wound pressure vessels are currently limited.

Embedding a nano-material-based sensor into the filament wound pressure vessel could perform the in-situ structural health monitoring to get the damage information [31,32,33,34]. MWCNTs can provide electrical percolation at a very low concentration, because of their high aspect ratio and highly conductive properties [35]. These outstanding properties make MWCNT a promising candidate for nanoelectronic sensors [36,37]. The present study therefore proposes a new resistance-based MWCNT sensor that can in-situ measure the damages in the composite skin of the composite pressure vessel. Compared with the traditional approaches of introducing CNTs into epoxy matrices for in-situ monitoring composite materials, this technique is easier to perform and also has the potential for industrial scale-up. The sensing ability and the reliability of the developed MWCNT sensor in various load conditions are firstly investigated. In-situ monitoring of the full-scale pressure vessels under hydraulic fatigue cycling and pressurization test are performed. The monitoring results are compared with the strain measured by the strain gauges, in order to demonstrate the validity of the MWCNT sensor.

## 2. Materials, Manufacturing, and Test Methods

### 2.1. Manufacturing and Reliability Test of the MWCNT Sensor

**Figure 1:**
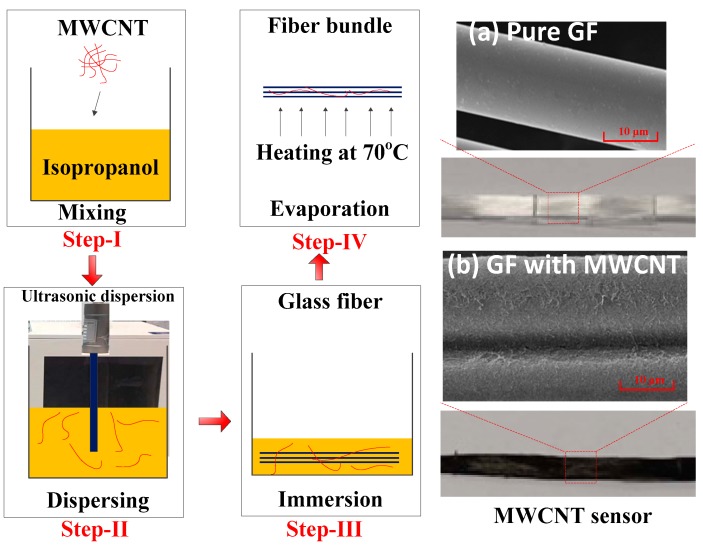
The manufacture processing of multi-walled carbon nanotube (MWCNT) sensor and its microcosmic appearance on a glass fiber (GF) bundle surface: (**a**) is the pure GF surface, while (**b**) is the GF fiber with MWCNT.

Figure 1 shows the manufacture processing of the MWCNT sensor. In detail, the sensor is comprised of the glass fiber-bundle with coated MWCNT on the surface. The physical vapor deposition (PVD) method was adopted to deposit the MWCNT on the glass fiber surface. The first step in the PVD method is to prepare the MWCNT solution. MWCNT (-COOH functionalized, Outer diameter >50 nm, length <10 μm, and purity >98 wt %; Time Nano Technologies, Inc., Chengdu, China) was added to an aqueous surfactant solution and was treated with an ultrasonic processor at a constant output power of 300 W. The aqueous surfactant solution was prepared by dissolving sodium dodecyl sulfate (SDS; Analytical reagent >88%; General reagent Inc., Shanghai, China) in deionized water. In the second step, a glass fiber-bundle was immersed in the prepared MWCNT solution for five cycles. Each cycle requires the bundle to be in the solution for 5 s and heated at 105 °C for 5 min in order to evaporate the deionized water and deposit the MWCNT on fiber surface. The concentration of the prepared MWCNT solution was N = 6.25 mg/ml, and the content of MWCNT on the glass fiber surface was 0.0238 mg/mm. The scanning electron microscope (SEM; EVO MA 15, Zeiss, Oberkochen, Germany) observations of the MWCNT sensor are presented in Figure 1a,b. As can be seen in Figure 1b, the MWCNT was fully coated on the glass fiber-bundle. In the following monitoring work, the resistance of the manufactured MWCNT sensor was recorded by a Keithley 2700 programmable electrometer.

To verify the reliability of the MWCNT sensor, we tested the sensing ability of the sensor in the short beam shear, flexural, tensile, and fatigue test of a composite laminates, before applying it in a real filament wound pressure vessel. In the mentioned mechanical tests, the MWCNT sensor was embedded into the GF/epoxy composite laminate by vacuum-assisted resin transfer molding (VARTM) processing. The details of the VARTM processing can be found in the literature [38,39]. The mechanical tests were accomplished using INSTRON-4505 servo-electric testing machine. To evaluate the through-the-thickness properties of the sensor in the composite laminates, three cases with the sensor in the upper, middle, and bottom layer in the laminates were considered in the short beam shear and flexural test. In the tensile and fatigue tests, the MWCNT sensor was embedded in the middle layer of the laminates. During the mentioned mechanical tests, the resistance of the MWCNT sensor was recorded simultaneously [40,41].

### 2.2. Manufacturing of the Filament Wound Pressure Vessel with an Embedded MWCNT Sensor

**Figure 2:**
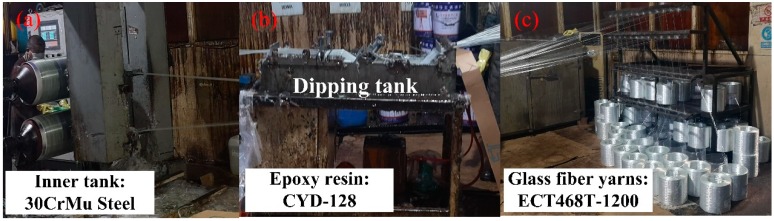
The manufacture processing of the filament wound composite pressure vessel: (**a**) is the rolling tank, (**b**) is the dipping tank, and (**c**) is the glass fiber yarns.

The filament wound pressure vessel was manufactured in Shanghai Ronghua High-Pressure Vessel Co, Ltd, Shanghai, China. It was made by filament winding around a 30CrMo steel inner tank. The manufacture processing of the filament wound composite pressure vessels is shown in Figure 2. The dimensions of the inner metal tank are as follows: the total length is 750 mm, and the diameter of the cylindrical and the hemispheric head section is 325 mm. The wall thickness of the tank is 5 mm with a design pressure of 20 MPa. The reinforced material in the composites skin is glass fiber yarn (type: ECT468T-1200, Changsheng Co., LTD, Hubei, China), with an average single fiber diameter of 20 μm. The yarns are bound on the vessel surface after going through the dipping tank. The polymer matrix in the composite skin is vinyl ester resin that can be cured at room temperature with a hardening and accelerating agent. The hardening agent is methyl ethyl ketone peroxide (MEKP), and the accelerating agent is dimethylaniline (DTLL). The amount of resin is controlled by a scraper system, to make sure the filaments are completely wetted. During winding, the excess resin is removed from the vessel surface, in order to ensure a high fiber volume ratio. The layup for the composite overwrap was [90°_10_/(±15°)_20_/90°_10_] during the winding process, and the pre-tension of yarn was kept constant at 40 N for all of the windings.

**Figure 3:**
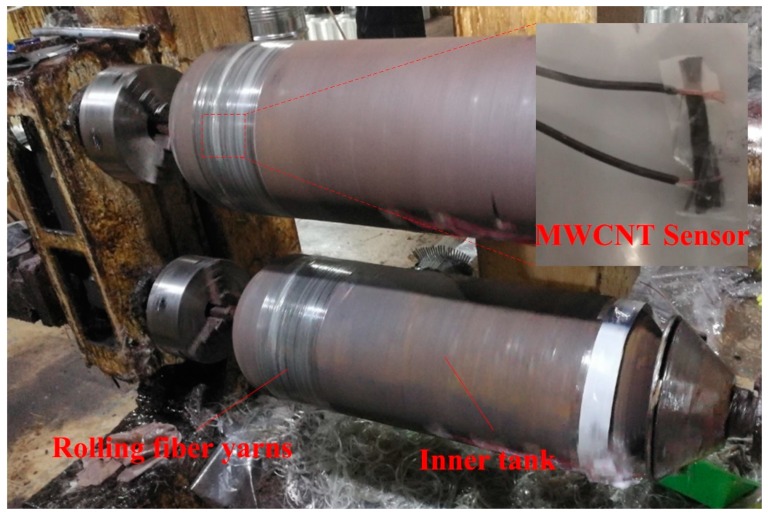
The MWCNT sensor is embedded in the middle thickness of the composite skin during the filament winding process.

**Figure 4:**
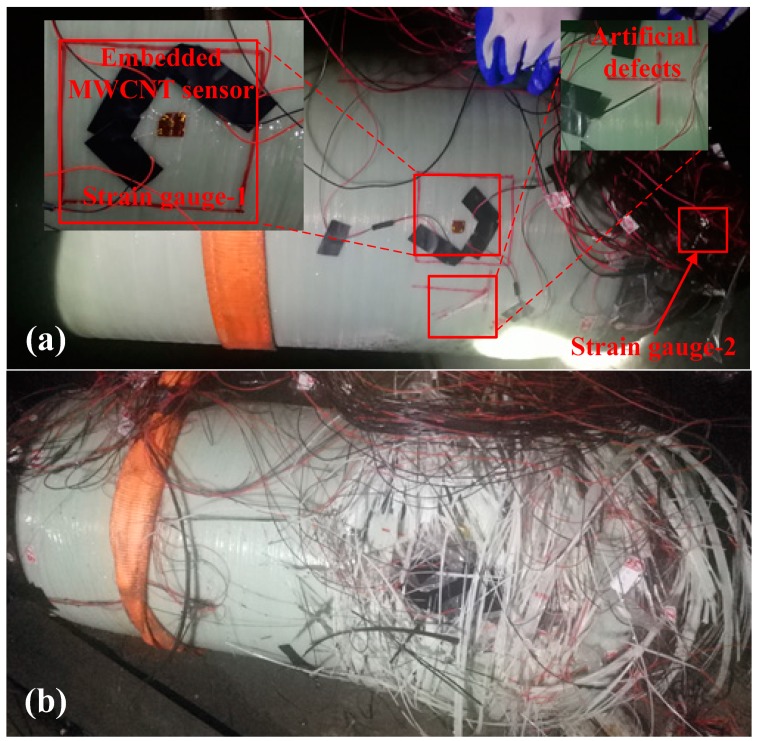
Pressure vessels before and after bursting: (**a**) before bursting, (**b**) after bursting.

The developed MWCNT sensors with an active length of 20 mm were embedded in the middle thickness of the composite layer, as shown in Figure 3. Two MWCNT sensors, with the direction along the axial and the circumferential of the pressure vessel were embedded, respectively. Moreover, to verify the sensing ability of the MWCNT sensor, two strain sensors were also used in the filament wound pressure vessel. The distance between one head of the pressure vessel and the embedded MWCNT sensor was 170 mm. One strain gauge was located on the composite skin of the pressure vessel (strain gauge-1), and it had the same location as the embedded MWCNT sensor. The other strain gauge (strain gauge-2) was located on the metal cylindrical section between the head and the composite overwrap, as shown in Figure 4a.

### 2.3. In-Situ Monitoring of Filament Wound Pressure Vessel under Hydraulic Fatigue Cycling and Pressurization

**Figure 5:**
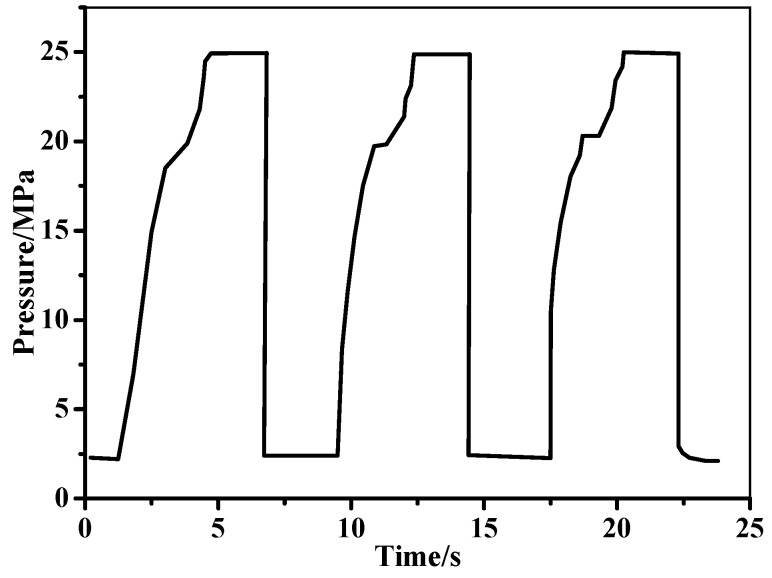
Parameters in hydraulic fatigue cycling.

The resistance and strain of the filament wound pressure vessel hydraulic fatigue cycling and pressurization were recorded by the MWCNT and strain sensors, respectively. The hydraulic fatigue cycling parameters were set as in Figure 5, and they are detailed in Table 1. The maximum/minimum pressures in one cycle in the hydraulic fatigue pressure test ranged from 2 to 25 MPa. Pressure was applied to vessels at a rate of 7.8 cycles/min. In the pressurization, the vessel was pressurized at a rate of 0.1 MPa/s, and the experiments were interrupted until the pressure was completely blasting. An artificial defect was made on the pressure vessel to make the vessel blasting at the special location. The artificial defect was a crack that was made by a low-speed diamond saw blade cutting machine. The depth and length of the crack was 6 mm and 55 mm, respectively. The crack was located 70 mm from the head, as marked in Figure 4a. As a result of the damage morphology (shown in Figure 4b), the artificial defect on the vessel also avoided the bursting position coincidence with the sensors, thus ensuring that the sensors could sense the whole damage of the filament wound pressure vessel. Two filament wound pressure vessels were used in the experiments. One pressure vessel was firstly tested in the fatigue test for 5700 cycles, and then was tested to the burst pressure directly. The other vessel was tested in the pressurization as the reference specimen. During all of the experiments, the resistance of the MWCNT sensor and the strain were recorded by the aforementioned technologies, respectively.

## 3. Sensing Ability of MWCNT Sensor in Various Load Conditions

The filament wound pressure vessel generally works in a complex working environment [8,42,43]. Given the uncertainties of the MWCNT sensor in the working environment, in this section, its reliability is considered in the short beam shear, flexural, tensile, and fatigue tests, before applying the sensor in the composite skin of a pressure vessel.

### 3.1. In-Situ Monitoring by the MWCNT Sensor in the Short Beam Shear and Flexural Tests

**Figure 6:**
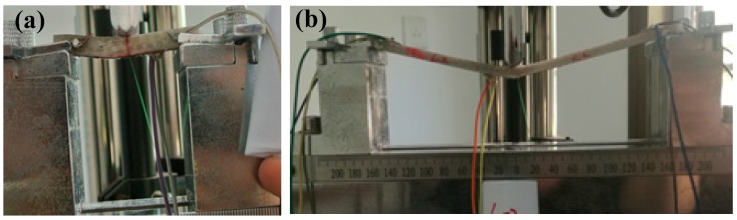
Experimental setup of the experiments with simultaneously resistance recording: (**a**) short beam shear test; (**b**) three-point-bending test.

In the short beam shear test, the dimension of the specimens is 50 × 14 × 6.2 mm^3^ with the span of 40 mm. In the flexural test, the dimension of the specimens is 180 × 15 × 6.2 mm^3^ with the span of 160 mm. The experiments are carried out in the three-point-bending mode with a cross-head loading speed of 2 mm/min. The radius of the cross head is 10 mm. The composite laminates are made of a unidirectional glass fiber reinforced epoxy matrix with the stacking sequence of [0°]_30_. The MWCNT sensor is located between the 1/2, 15/16, and 29/30 layers in the laminates, respectively. During the test, the sensor is connected with the Keithley 2700 programmable electrometer by a conductive silver paint, as shown in the experimental setup in Figure 6. The conductive silver paint is located on both ends of the specimens with the painting width of 2 mm, to ensure full contact between the wire and the sensor in the measurement.

**Figure 7:**
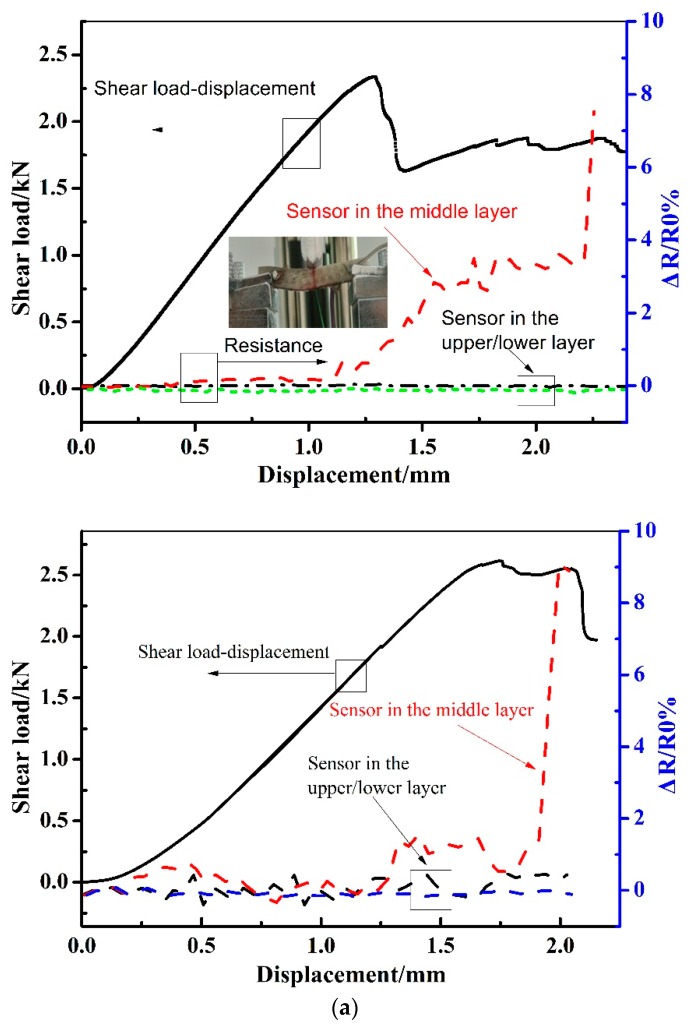

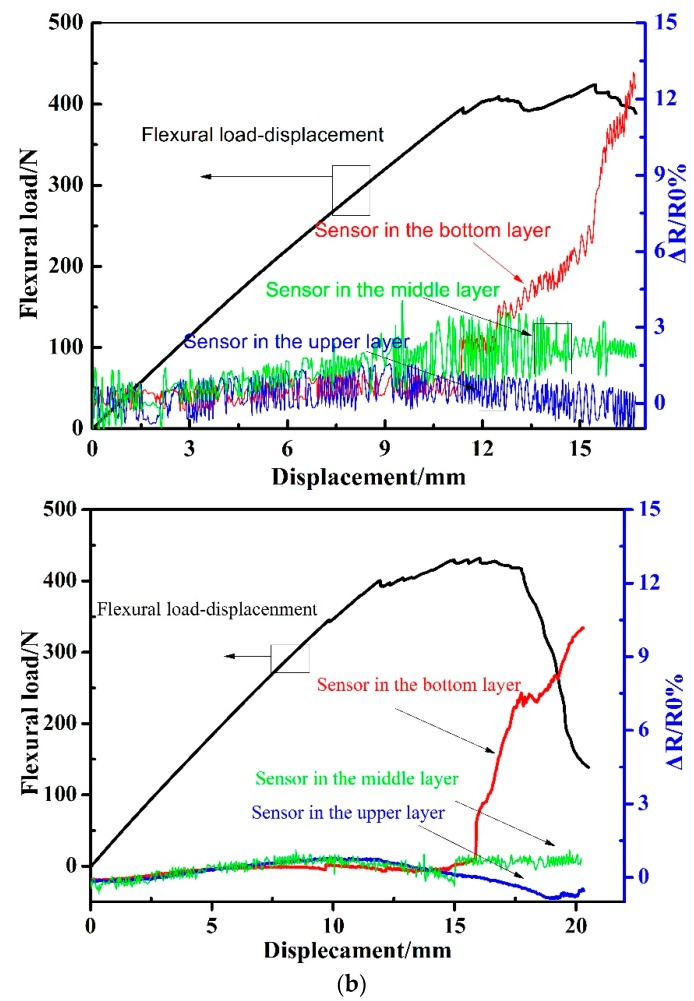
Resistance change of the MWCNT sensor in short beam shear and flexural tests of the unidirectional composite laminates. The repeat curve is also given in the figure. (**a**) Short beam shear and the parallel sample. (**b**) Flexural and the parallel sample.

Figure 7 shows the load–displacement curves together with the corresponding resistances recorded by the MWCNT sensors embedded into the composite laminates. To verify the repeatable performance of the MWCNT sensor in the short beam shear and flexural test, we also give the test results of a parallel sample in Figure 7. As can be seen in the figure, all of the samples have roughly the same electrical resistance change. In the figure, the relative resistance Δ*R*/*R*0 is determined by the following:

(1) $$\Delta R/R0=\frac{R-R0}{R0}\times 100\%$$

where *R* is the test resistance during loading, while *R*0 is the initial resistance before the mechanical tests. As can be seen in Figure 7a, the relative resistance tested by the upper/lower sensor keeps steady at 0 with the increasing of load–displacement curve, until the test finished. As a comparison, the relative resistance tested by the sensor in the middle thickness of the laminates remained at 0, before the shear load reaches to the maximum at 2.3 kN, and it starts increasing with the displacement when the shear delamination happens after the maximum shear load. It can be seen that the sensors in the upper/lower layer showed no difference in electrical resistance, while only the sensor in the middle showed a difference in resistance. This is because the damages formed in the composite laminates under a short beam shear load are the shear delamination that is located in the middle layer of the specimens. The sharp increase of relative resistance at a displacement of 2.25 mm indicates the complete delamination in the middle thickness of the laminates in the shear test. The above results indicate that the MWCNT sensor located in the middle thickness could effectively monitor the shear damage of the composite laminates. The resistance change is because of the shear deformation that severs the electrical paths, and the contact resistance is substituted by the tunnel resistance between the different MWCNTs in the sensor [30,37]. As can be seen in the flexural test in Figure 7b, the relative resistance of the MWCNT sensor in the bottom layer of the laminates could sense the flexural load–displacement well, and it shows a sharp increase when the specimen is completely damaged under the flexural load. As known, the damage region of the specimen after the flexural test can be divided into three parts, namely: compressive damage region on contact surface, fiber tensile breaking region in bottom surface, and crack region inner the specimen. As the deformation in the bottom side of the laminates in the flexural test is the largest, it causes the largest deformation, which can be easily sensed by the MWCNT sensor located in the bottom surface. The above results indicate that the developed MWCNT sensor could sense the shear load and the flexural load in the laminates well when the sensor is located in a suitable location.

### 3.2. In-Situ Monitoring by the MWCNT Sensor in the Tensile and Fatigue Test of Composite Laminates

**Figure 8:**
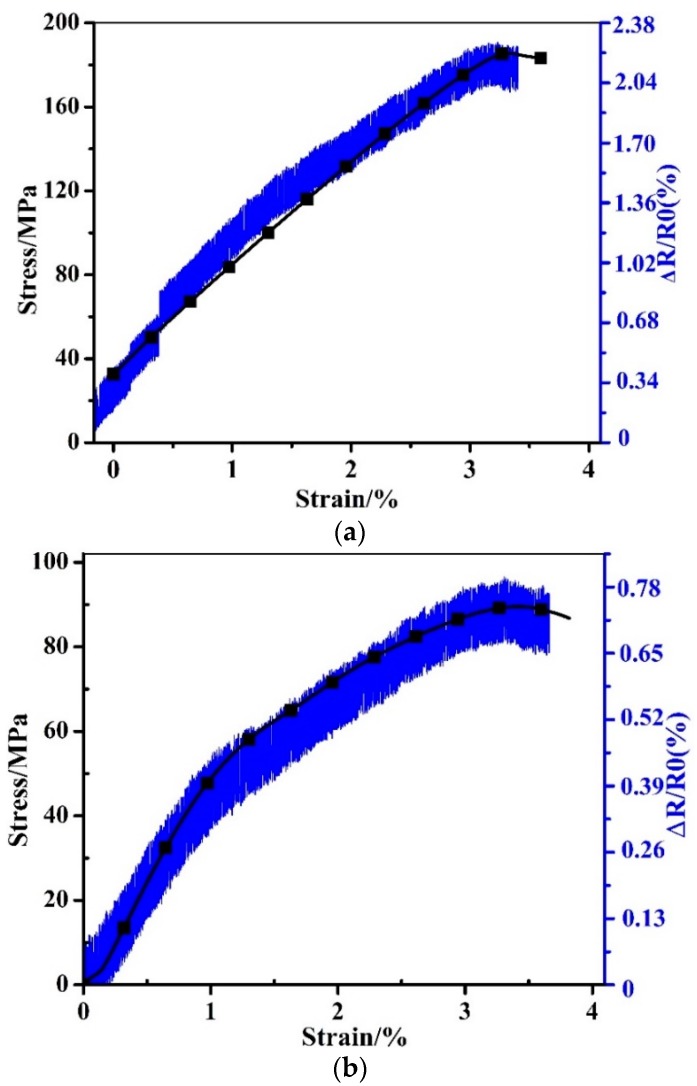
Resistance–loading time in the tensile tests. (**a**) Longitudinal resistance vs. loading strain. (**b**) Transverse resistance vs. loading strain.

In the tensile test, the MWCNT sensor was located in the longitudinal and transverse direction of a woven glass fabric reinforced epoxy composite laminate with stacking sequence of [0°/90°]_11_, respectively. The sensing length in the longitudinal resistance test in Figure 8a is 200 mm, while it is 20 mm in the transverse resistance test in Figure 8b. As shown in Figure 8a, the longitudinal resistance shows an increased tendency with the increase of the tensile stress, until the test is finished at a strain of 3.5%. When the tensile stress increases from 0 to 185 MPa in the test, the relative resistance of the MWCNT sensor increases from 0% to 2.1%, simultaneously. In the case of the transverse resistance in Figure 8b, the relative resistance increases from 0% to 0.78% with the increasing tensile stress, and the tendency of the stress and the resistance as a function of the loading time matches quite well. The above results indicate that the applied MWCNT sensor could also monitor the tensile stress of the composites laminates well in both the longitudinal and transverse direction of a woven composite laminate. As discussed, the macroscopic resistance changes of the embedded MWCNT sensor are due to the fact that the conductive networks can be broken up by the appearance of damage. It should be noted that five specimens were tested in the above mechanical tests. In Figure 8, the average *ΔR/R*0 of the longitudinal resistance is 2.24 ± 0.13, while it is 0.70 ± 0.05 for the transverse resistance. The average test results, together with their standard deviations, of the composite laminates with and without MWCNT are listed in Table 2. As can be seen in the table, the mechanical performances of the specimens with and without MWCNT are pretty close, which means that the additional sensor did not harm the mechanical properties of the composites.

**Figure 9:**
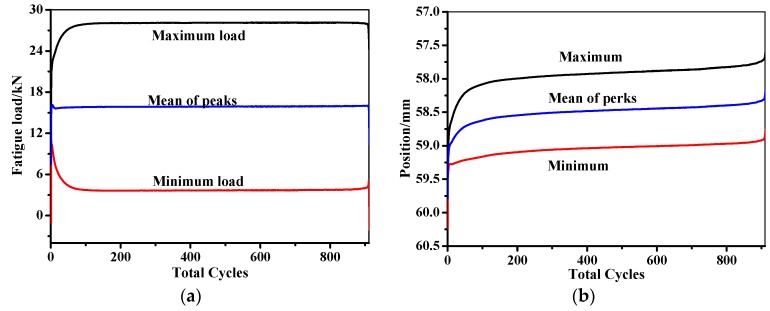
Parameters in the fatigue test. (**a**) Fatigue load–cycles. (**b**) Fatigue position–cycles.

**Figure 10:**
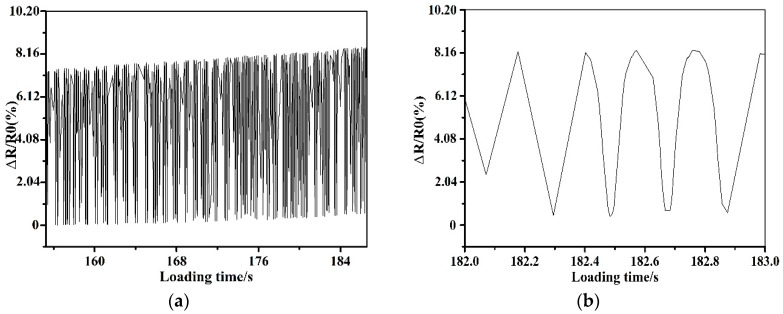
Resistance change of the MWCNT sensor during fatigue. (**a**) Resistance–loading time. (**b**) The enlarged drawing curves.

The composite laminates adopted in the fatigue test have the same dimension and stacking sequence as the specimens in the tensile test. In the fatigue test, the maximum and minimum load is set as 3 and 30 kN, respectively. The stress ratio, *R*, is defined as the minimum load to the maximum load in the fatigue, and is kept constant at 0.1, with a loading frequency of 5 Hz. The displacement fluctuation range in the fatigue test is 1 mm, as illustrated in Figure 9. A maximum of 1000 cycles are performed in the fatigue test. The resistance changes of the MWCNT sensor, together with the enlarged drawing curve when the loading time ranges from 182 to 183 s during the fatigue test, are shown in Figure 10. As can be seen in the figure, the recorded resistance matches the load tendency well, even when the composite laminates undergo 1000-cycle’s fatigue. The results verified that the mentioned monitoring method by the developed MWCNT sensor also has the potential for in-situ sensing the fatigue stress of the composite skins in the filament wound pressure vessels.

## 4. Applying the MWCNT Sensor in Composite Pressure Vessel

### 4.1. In-Situ Monitoring of the Pressure Vessel during Hydraulic Fatigue Cycling

**Figure 11:**
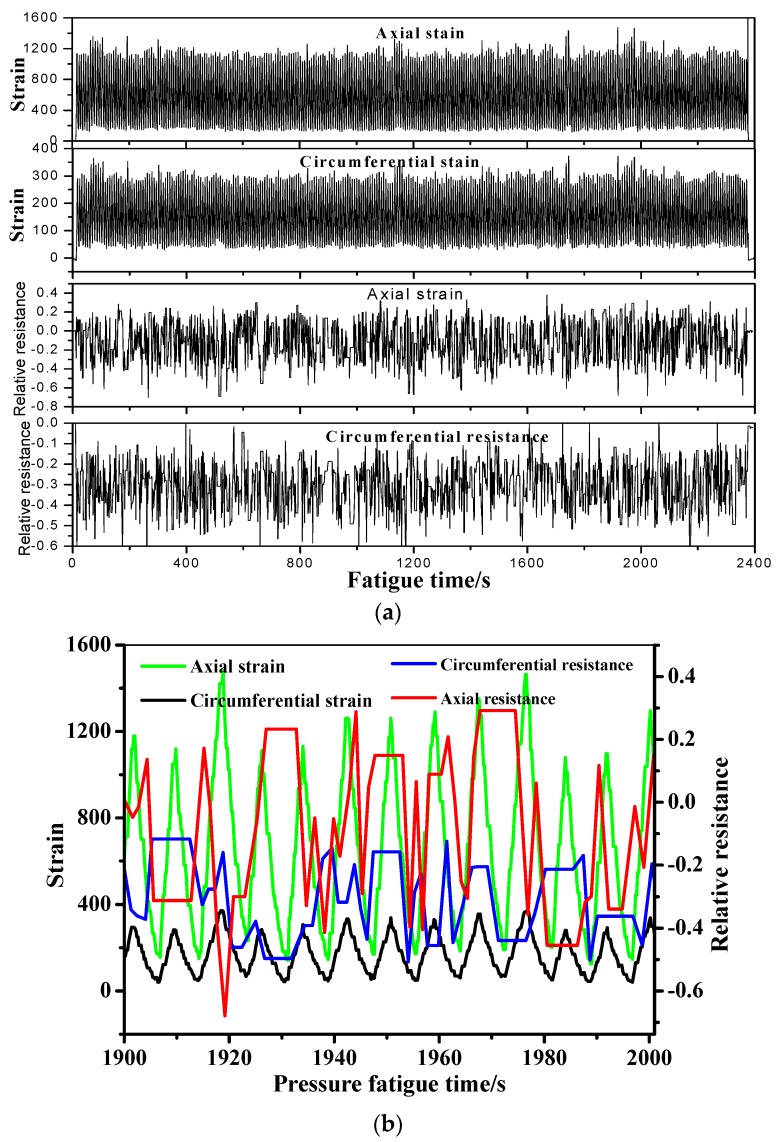
Monitoring results by the MWCNT sensor and the stain gauge when the filament wound pressure vessel is in 2100- to 2400-fatigue cycling. The strain is measured by strain gauge-2 in Figure 4. (**a**) Resistance, the stain as a function of the fatigue time. (**b**) The enlarged drawing curves.

**Figure 12:**
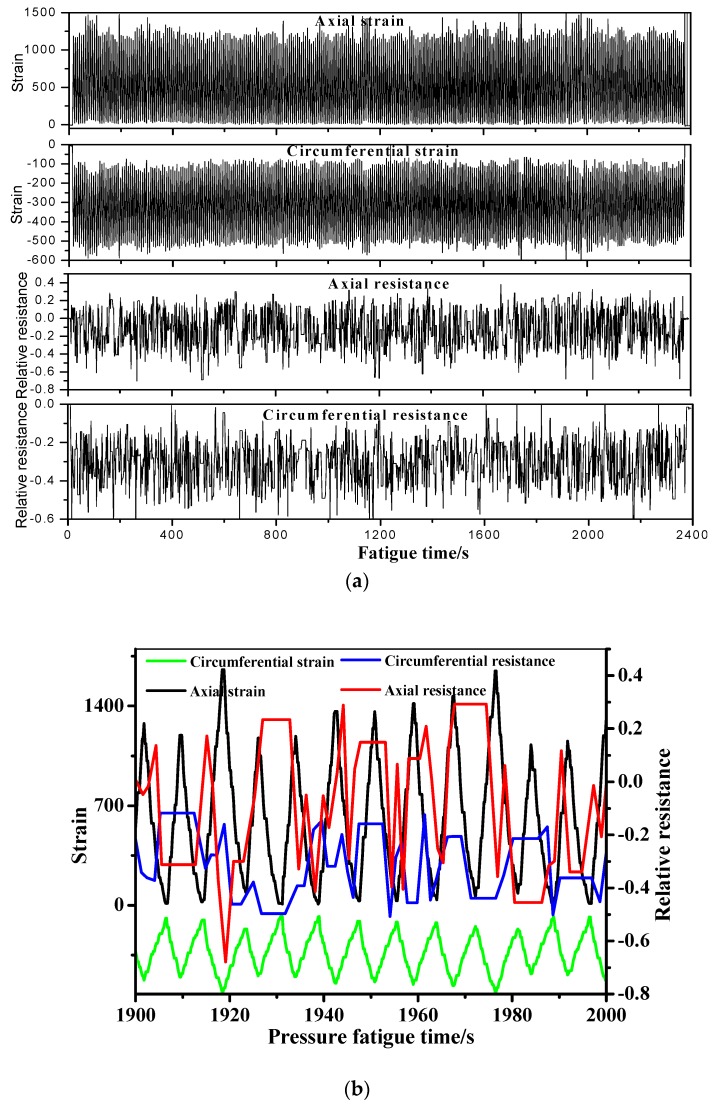
The monitoring results of the developed MWCNT sensor and the stain when the filament wound pressure vessel is in 2100- to 2400-cycles loading. The strain is measured by strain gauge-1 in Figure 4. (**a**) Resistance, the stain as the function of fatigue time. (**b**) The enlarged drawing curves.

Figure 11a shows the recorded axial and circumferential relative resistance and strain as a function of the pressure fatigue time. The hydraulic fatigue is between 2100 to 2400 cycles. To show the detailed information of the monitoring signals, the enlarged drawing curve between 1900 to 2000 s is shown in Figure 11b. For the pressure vessel in pressurization, the axial strain is generally larger than the circumferential strain [9]. The strain tendency in Figure 11b also verifies this conclusion. In the fatigue test, the minimum and maximum axial/circumferential strain fluctuate from 179 to 1295 and 48 to 334, respectively. The relative resistance of the MWCNT sensor varies from −0.31 to 0.235 and −0.435 to −0.204, in accordance with the fatigue parameters in Figure 5. It should be noted that the pressure holding time is also reflected by the small platform in the resistance–fatigue time relationship in Figure 11b. Because the MWCNT sensor is embedded in the middle thickness of the composite skin of the pressure vessel, we also compared the resistance signal with the strain measured by strain gauge-1 in Figure 4. Figure 12 illustrates the comparison of the results between the composite stain measured by strain gauge-1 and the relative resistance of the MWCNT sensor in the hydraulic fatigue cycling from 2100 to 2400 cycles. As can be seen in the figure, the axial and circumferential composite strain varies from 70 to 1468 and −546 to −105, respectively. Compared with the strain measured by strain gauge-2 on the inner tank, the axial/circumferential strain on the composite skin are larger in the same loading condition. This phenomenon is mainly due to the lower stiffness of the composite laminates. Meanwhile, the larger radius of the composite layer also leads to a larger stain than that of the inner tank under the same inner pressure. The tendency of the relative resistance matches the composite strain well, which further validates the feasibility of the developed MWCNT sensor applied in the in-situ monitoring of the filament wound pressure vessel.

**Figure 13:**
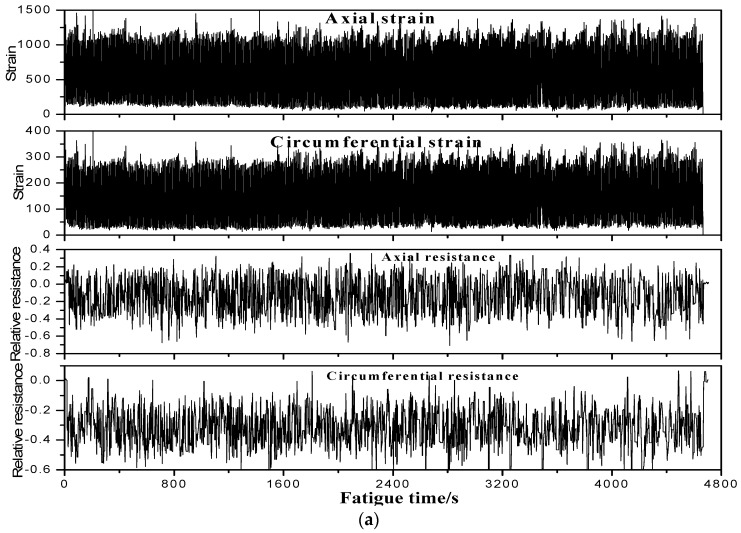

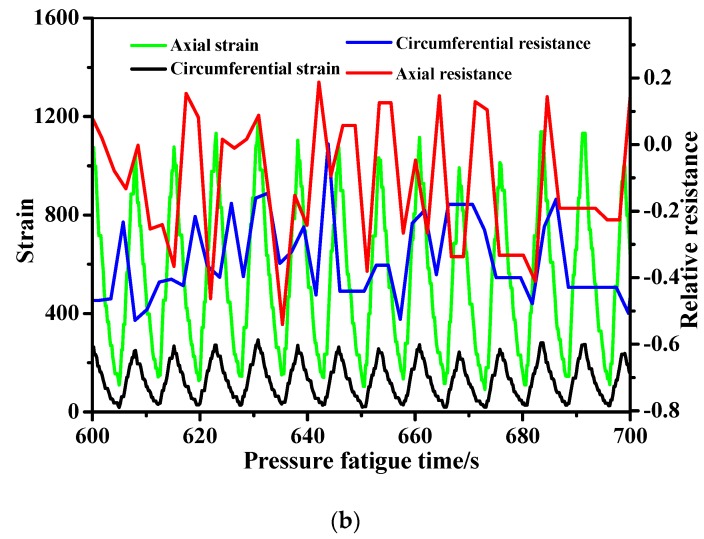
Monitoring results by the MWCNT sensor and the stain gauge when the filament wound pressure vessel is in 5100- to 5700-fatigue cycles. The strain was measured by strain gauge-2 in Figure 4. (**a**) Resistance, the stain as the function of fatigue time. (**b**) The enlarged drawing curves.

**Figure 14:**
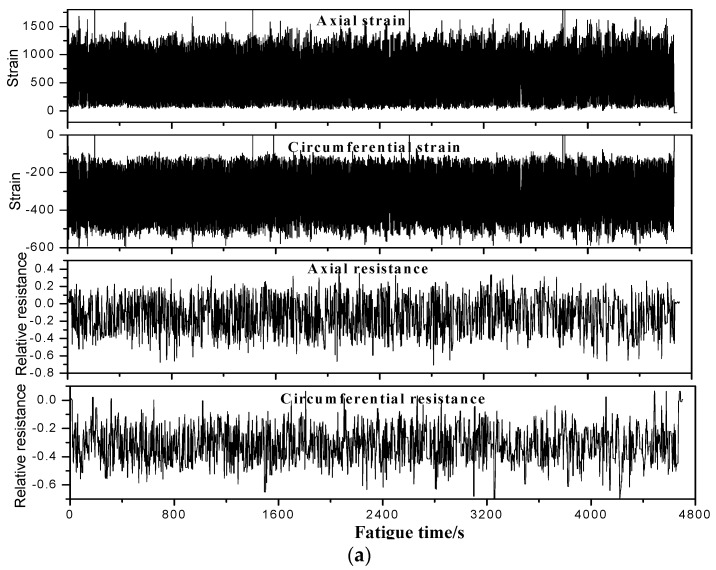

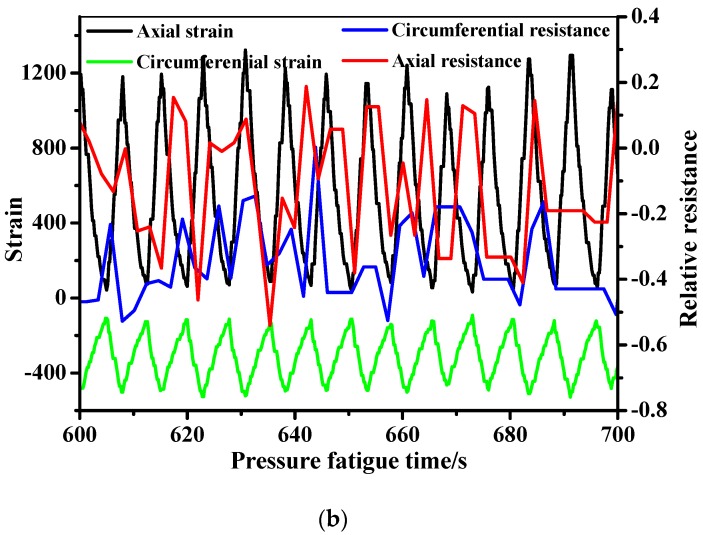
Monitoring results of the MWCNT sensor and the stain gauge when the filament wound pressure vessel is in 5100- to 5700-fatigue cycles. The strain was measured by strain gauge-1 in Figure 4. (**a**) Resistance, the stain as the function of fatigue time. (**b**) The enlarged drawing curves.

The in-situ monitoring results of the MWCNT sensor in the filament wound pressure vessel during hydraulic fatigue cycling up to 5700 cycles are shown in Figure 13 and Figure 14. In the figures, the sensing resistance is also compared with the strain of the metal tank and the composite skin in the axial and the circumferential direction of the vessel, respectively. The minimum/maximum relative resistance in the circumferential direction changes from −0.443 to −0.18, while it changes from −0.4 to 0.2 with the applied pressure in the vessel in the axial direction. For the stain in the metal tank, the circumferential/axial strain changes from 20 to 240 and 120 to 993, respectively. In the case of the stain measured by strain gauge-1 on the composite skin, the circumferential/axial strain changes from −505 to −115 and 74 to 1165, respectively. It should be noted that the signals from the MWCNT sensors do not appear to match the fatigue cycles in Figure 11, Figure 12, Figure 13 and Figure 14. This is because the tank/composite interface could interfere with the deformation transfer in the composite under fatigue. Thus, the deformation inner in the middle thickness of the composite skin in each fatigue cycle does not match the fatigue loading well. The complex damage mechanism of the composite skin of the pressure vessel under fatigue leads to the resulting data in Figure 11, Figure 12, Figure 13 and Figure 14. Table 3 summarizes the equivalence relationship between the resistance tested by the embedded MWCNT sensor and the two strain gauges in different directions. The relative resistance and strain listed in the table are calculated by the difference between the maximum and the minimum value, respectively. The relationship in Table 3 can be used to quantificationally evaluate the strain status in the metal tank and the composite layer when using the developed MWCNT sensor, to monitor the health status of the filament wound pressure vessel. For example, in the fatigue cycles between 2100 to 2400, the measured relative resistance (0.545) in the axial direction by the MWCNT sensor is equal to the strain of 1100 and 1398 measured by the strain gauges, respectively. Moreover, the strain of the two strain gauges as a function of fatigue cycles is listed in Table 4. As can been seen in the table, the residual strain is found for the pressure vessel after fatigue, and the residual strain of the composite skin is generally larger than that of the metal tank. The residual strain indicates that the filament wound pressure vessel is damaged in the hydraulic fatigue cycling. Therefore, in the following work, we also investigate a health vessel as the reference experiment.

### 4.2. In-Situ Monitoring of the Pressure Vessel during Pressurization

**Figure 15:**
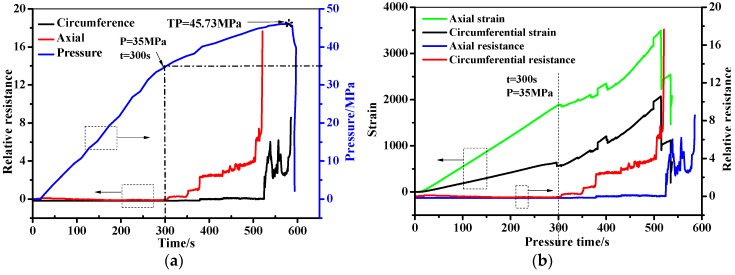
The in-situ monitoring of the filament wound pressure vessel (after hydraulic fatigue for 5700 cycles) during the blasting test. (**a**) Resistance, pressure–pressure time. (**b**) Resistance, strain–pressure time.

**Figure 16:**
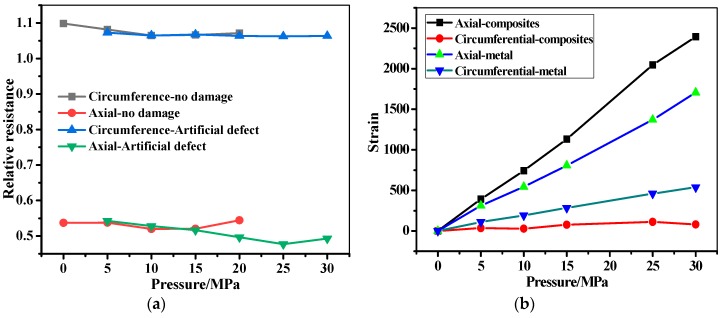
The in-situ monitoring of the filament wound pressure vessel (after hydraulic fatigue for 5700 cycles) before bursting. (**a**) Resistance change as a function of pressure. (**b**) Strain change as a function of pressure.

The filament wound pressure vessel after hydraulic fatigue for 5700 cycles was then used in the bursting test. The relative resistance, pressure, and the stain as a function of the pressure time are shown in Figure 15. As can be seen in Figure 15a, the pressure in the filament wound pressure vessel increases from 0 to 45.73 MPa, until the wound pressure vessel is burst sharply. The pressure–time curve shows a bilinear behavior at the knee point of P = 35 MPa and t = 300 s. The relative resistance firstly shows a steady tendency at 0 with the increase of pressure, until reaching the knee point of the pressure–time curve. Then, the relative resistance shows an increasing tendency with the pressure, until the bursting of the vessel at t = 600 s. Also, it should be noted that the signals fluctuated in the minoring process when the pressure ranged from 35 to 45.73 MPa, which is mainly due to the gradual damage feature of the composite skin [44,45,46]. The relative resistance is compared with the stain measured by strain gauge-2 in Figure 15b. As can be seen in the figure, the strain and the resistance have the same tendency. The axial/circumferential strain before 35 MPa in pressurization show a linear increase from 0 to 1887 and 616, respectively. The two stains then increased to 3502 and 2082 form 35 MPa to the burst pressure, respectively. It should be noted that the increment does not follow a linear tendency, and the fluctuation of the stain is found. The relative resistance and the stain of the filament wound pressure vessel after 5700 cycles’ fatigue as a function of the pressure in the range of 0 to 30 MPa are shown in Figure 16. In Figure 16a, the measured resistance by the MWCNT sensor in the filament wound pressure vessel without defects is also drawn. The resistance of the MWCNT sensor keeps a constant value, while the strain shows an increased tendency with the inner pressure of the filament wound pressure vessel. This phenomenon indicates that damages such as the delamination and/or fiber breaking in the composite skin of the pressure vessel before 30 MPa did not happen.

**Figure 17:**
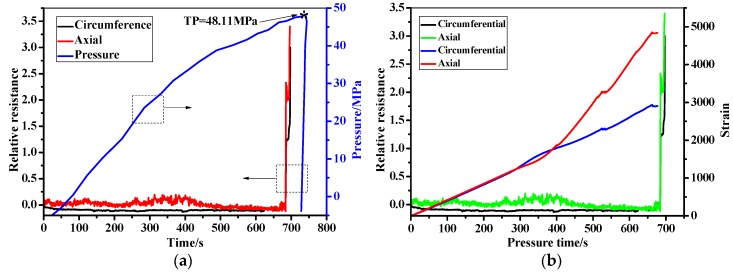
The in-situ monitoring of the health filament wound pressure vessel during pressurization. (**a**) Resistance, pressure–pressure time. (**b**) Resistance, strain–pressure time.

**Figure 18:**
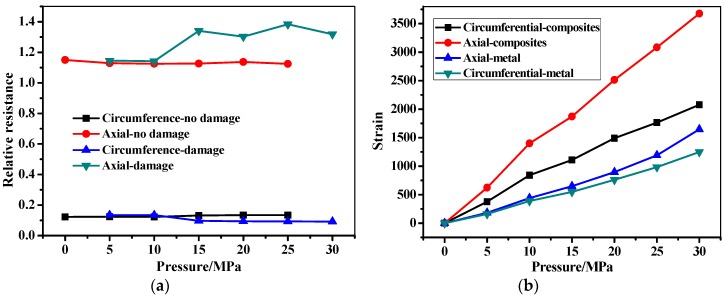
The in-situ monitoring of the health filament wound pressure vessel before bursting. (**a**) Resistance change as a function of pressure. (**b**) Strain change as a function of pressure.

Figure 17 and Figure 18 show the in-situ monitoring results of a health filament wound pressure vessel up until and before the burst pressure. The burst pressure of the health pressure vessel is 48.11 MPa, which is 2.38 MPa than that of the vessel after the fatigue for 5700 cycles. As discussed, this difference is also illustrated by the residual stain after the hydraulic fatigue listed in Table 3. The measured resistance signal shows a sharp increment when the pressure increases to the burst pressure. Unlike the test curve in Figure 15a, the resistance fluctuates before reaching the sharp increment point at 700 s. The relative resistance and the strain shown in Figure 17b illustrate the corresponding relationship between the two monitoring signals. In Figure 18, we also illustrate the in-situ monitoring result of the health filament wound pressure vessel up to 30 MPa in pressurization. The results indicate that the damage inner of the composite skin did not appear in the health pressure vessel under 30 MPa.

## 5. Conclusions

This paper mainly aims to study the in-situ monitoring of a filament wound pressure vessel under hydraulic fatigue cycling and pressurization, by the developed MWCNT sensor. The manufacturing and sensing performance of the MWCNT sensor are firstly discussed, and then the sensor is applied in a full-scale filament wound pressure vessel. The following conclusions can be drawn in this study:

- The developed MWCNT sensor could in-situ sense the health status of the composite laminates under a short beam shear and flexural tests. The shear damage inner, the middle thickness, and the larger deformation of the bottom surface in the specimens could lead to the resistance change of the sensor, respectively. In the tensile and fatigue test, the measured resistance signal also matches the mechanical behavior of the structure well.
- The MWCNT sensor could monitor the filament wound pressure vessel in the hydraulic fatigue cycling, and the equivalence relationship between the relative resistance and the strain is built in the fatigue test.
- In the bursting test, the relative resistance remains 0 before the pressure in the vessel reaches 30 MPa, which indicates that damages do not appear in the composite skin before 30 MP for a pressure vessel with burst pressure of 48.11 MPa. The changing tendency of the resistance signal matches the strain well, and the bursting of the pressure vessel can be reflected by the sharp increase of the resistance measured by the embedded MWCNT sensor.

## Figures and Tables

**Table 1:** Parameters of the hydraulic fatigue cycling.

Item	Parameter	Item	Parameter
Cycle times	5700	Rising pressure time	3.1 s
Maximum pressure	25 Mpa	Max pressure holding time	2 s
Pressure drop time	0.6 s	Minimum pressure	2 s
Min pressure holding time	2 s	Cycling rate	7.8
Temperature	11.2 °C	Media	water

**Table 2:** The mechanical performance of the composites laminates before and after embedding the multi-walled carbon nanotube (MWCNT) sensors in different mechanical tests.

Specimens	Failure Load in Short Beam Shear/N	Failure Load in Flexural/N	Tensile Strength/Mpa
Without sensor	2318 ± 10.26	421.52 ± 0.86	186.43 ± 8.29
With sensor	2330 ± 7.68	424.22 ± 0.86	185.66 ± 5.23

**Table 3:** The equivalence relationship between the resistances tested by the embedded MWCNT sensor and the strain gauges.

Fatigue Cycle	Test Direction	Strain Gauge-2	Strain Gauge-1	Relative Resistance
2100–2400	axial	1100	1398	0.545
	circumferential	286	441	0.639
5100–5700	axial	873	1091	0.466
	circumferential	220	390	0.263

**Table 4:** The residual strain as a function of pressure fatigue cycles.

Pressure Fatigue Cycles	Strain Gauge-1		Strain Gauge-2	
	Axial	Circumferential	Axial	Circumferential
300	42.4	14.5	8.2	3
1500	25.6	7.8	7.8	6.4
1800	23.3	6.1	8.2	6.4
2100	25.9	8.9	4.8	8.8
2400	14.2	14.7	6.6	6
2700	15.3	11.9	8	10.4
3000	20.6	8.9	2.3	3.3
3300	27.4	11.5	13.5	12
5100	36.2	6.4	6.5	6.5
5700	13.1	31.7	71	15.8
